# Molecules, microbes, and function: synchronizing depth-resolved molecular and microbial time series at BATS

**DOI:** 10.1128/msystems.00014-26

**Published:** 2026-03-06

**Authors:** Michael S. Rappé

**Affiliations:** 1 Hawai‘i Institute of Marine Biology, School of Ocean and Earth Science and Technology, University of Hawai‘i at Mānoa 53738, Kāne'ohe, Hawai‘i, USA; CNRS Delegation Alpes, Villeurbanne, Rhône-Alpes, France

**Keywords:** dissolved organic matter (DOM), microbial communities, time series, functional redundancy, oligotrophic ocean, wavelet analysis

## Abstract

The marine dissolved organic matter (DOM) pool is one of Earth’s largest carbon reservoirs and a critical regulator of global carbon flux, yet the microbe-molecule interactions governing it remain largely unresolved. In a significant advancement, McParland and colleagues integrate a 3-year, depth-resolved molecular time series of DOM with parallel taxonomic characterization of prokaryoplankton at the Bermuda Atlantic Time-series Study site to examine the coupling between community composition and DOM molecules (E. L. McParland, F. Wittmers, L. M. Bolaños, C. A. Carlson, et al., mSystems 11:e01540-25, 2026, https://doi.org/10.1128/msystems.01540-25). Their analysis reveals high temporal coordination, with approximately 40% of both DOM molecules and microbial taxa exhibiting 12-month periodicities. However, interannual patterns in DOM composition appear more predictable than that of microbial communities, suggesting that functional redundancy, conferred through core metabolic pathways shared across taxa, may act as a stabilizing force for ocean chemistry. By highlighting the endurance of these functional roles, McParland and colleagues provide a framework for incorporating microbial processes into more mechanistic and predictable biogeochemical models.

## COMMENTARY

Marine microbes are one of the primary architects of the global carbon cycle (1), yet the cryptic nature of the dissolved organic matter (DOM)-microbe network has long limited the ability to predict how changes in one pool alter the composition of the other (2, 3). While microbial communities have been characterized at high resolution for over a decade, parallel observations of the specific molecules they cycle at a similar resolution have remained sparse. McParland and colleagues address this gap by presenting a parallel time series of untargeted DOM chemistry and prokaryoplankton taxonomy from the surface down to 200 m in the upper mesopelagic zone of the water column at the Bermuda Atlantic Time-series Study (BATS) site in the Sargasso Sea (4). To navigate the complexity of comparing fundamentally different units of measurement (16S rRNA gene amplicon sequences and mass spectrometry features), the authors utilized wavelet analysis to decompose temporal dynamics into the frequency domain. This approach allows recurring structure to be compared without assuming direct correspondence, identifies dominant recurring cycles (such as annual), and quantifies how well a signal fits the dominant cycle over the full sampling period (5, 6).

While the temporal and vertical structure of the microbial community and bulk DOM dynamics at BATS are among the most well studied of any planktonic marine system (7, 8), much less is known about how individual DOM molecules behave across depth and season. This study reveals that both the microbial community and individually identified molecules within the DOM pool are highly structured by depth-specific seasonality. By employing median power as an estimate of “best fit” for the 12-month cycle, the authors found that the DOM pool exhibits stronger recurring patterns between years than the prokaryoplankton community. Rather than suggesting the microbial community is inherently unstable, this finding indicates that the chemical output of the ecosystem is more predictable between years than the specific microbial taxa that are present at any given time. The persistence of these chemical patterns in the face of higher taxonomic variability is the core evidence for functional redundancy, wherein conserved biochemical pathways are distributed across a taxonomically flexible array of microbial assemblages. This indicates that marine ecosystems can tolerate some taxonomic reorganization without altering their chemical output, provided that the underlying genetic potential for core metabolic capacities is retained.

To explore the biological drivers of this chemical stasis, the authors putatively identified four seasonal exometabolites, including gonyol, glucose 6-sulfate, succinate, and trehalose. By tracking these molecules across the 3-year study period and querying historical BATS metagenomes for their associated metabolic pathways, they revealed that core microbial metabolisms are the most important predictors of DOM composition. Using the contribution evenness metric to quantify metabolic redundancy, McParland and colleagues found that molecules involved in widely conserved pathways, such as the citric acid cycle for succinate, showed high redundancy and interannual stability. Conversely, molecules like trehalose that are likely utilized by a narrower subset of the community contained lower redundancy yet still maintained consistent seasonal periodicity. This suggests that the predictability of biogeochemical fluxes depends on the distribution of core metabolic capabilities within a community rather than simple taxonomic presence.

While this study represents a clear step forward from bulk DOM measures to molecular-level insight in space and time, it also highlights the ongoing challenges of environmental metabolomics. For example, solid-phase extraction is known to bias toward smaller, more recalcitrant molecules, and many metabolic pathways for observed molecules remain unannotated in genomic databases (9, 10). Furthermore, the comparison of taxonomic stasis to chemical stasis requires technical caution, as these results emerge from very different analytical pipelines. This mismatch is difficult to resolve with current methodology. Future research must bridge this gap and move from static inventories of standing stocks toward active rate measurements and isotope tracing (10).

By illustrating how core metabolic pathways may endure despite shifts in taxonomic composition, McParland and colleagues provide a compelling conceptual advance. Their findings suggest that while specific microbial assemblages may be transient, the consistency of their metabolic roles leaves a stable chemical imprint, rendering the ocean’s molecular pool a persistent memory of microbial life. As anthropogenic stressors drive shifts in community structures, understanding the limits of this functional redundancy buffer will be critical for predicting future changes in the ocean’s organic carbon cycle.
